# Co-occurring protein phosphorylation are functionally associated

**DOI:** 10.1371/journal.pcbi.1005502

**Published:** 2017-05-01

**Authors:** Ying Li, Xueya Zhou, Zichao Zhai, Tingting Li

**Affiliations:** 1 Department of Biomedical Informatics, School of Basic Medical Sciences, Peking University Health Science Center, Beijing, China; 2 Department of Psychiatry and Centre for Genomic Sciences, Li Ka Shing Faculty of Medicine, The University of Hong Kong, Hong Kong SAR, China; 3 Institute of Systems Biomedicine, School of Basic Medical Sciences, Peking University Health Science Center, Beijing, China; Indiana University, UNITED STATES

## Abstract

Post-translational modifications (PTMs) add a further layer of complexity to the proteome and regulate a wide range of cellular protein functions. With the increasing number of known PTM sites, it becomes imperative to understand their functional interplays. In this study, we proposed a novel analytical strategy to explore functional relationships between PTM sites by testing their tendency to be modified together (co-occurrence) under the same condition, and applied it to proteome-wide human phosphorylation data collected under 88 different laboratory or physiological conditions. Co-occurring phosphorylation occurs significantly more frequently than randomly expected and include many known examples of cross-talk or functional connections. Such pairs, either within the same phosphoprotein or between interacting partners, are more likely to be in sequence or structural proximity, be phosphorylated by the same kinases, participate in similar biological processes, and show residue co-evolution across vertebrates. In addition, we also found that their co-occurrence states tend to be conserved in orthologous phosphosites in the mouse proteome. Together, our results support that the co-occurring phosphorylation are functionally associated. Comparison with existing methods further suggests that co-occurrence analysis can be a useful complement to uncover novel functional associations between PTM sites.

## Author summary

In addition to gene expression and translation control, post-translational modifications (PTMs) represent another level to regulate proteins functions. Different PTM sites within a protein usually co-operate to fulfill their functional roles. Recent advances in high-throughput mass spectrometry (MS) technologies have facilitated the proteome-wide identification of PTM sites, giving rise to both challenge and opportunity to understand their functional relationships. Previously, several data mining approaches have been developed to explore the global PTM interplays. In this study, we proposed to infer functional associations between PTM sites from the correlation of their modification status across many biological conditions, which was not exploited before. In practice, we tested if a pair of sites are modified together under the same condition significantly more often than expected (co-occurrence). As a proof of principle, we applied this analytical strategy to human phosphorylation because we could collect data sets of proteome-wide coverage under 88 different conditions. We demonstrated that sites with co-occurring phosphorylation status are functionally associated from several lines of evidence. The co-occurrence analysis can also uncover functionally connected phosphosites with clear biological evidence which are missed by other approaches. With increasing proteome-wide data for other types of PTMs under different conditions, the co-occurrence analysis can be integrated with other methods to identify novel PTM associations.

## Introduction

In addition to gene expression and translation, post-translational modification (PTM) represent another level of regulation that expands the functional capacity of proteins. It play a crucial role in a plethora of biological processes including regulation of gene expression [1], modulation of enzymatic activity [2, 3], and control of protein-protein interaction (PPI) [4]. More than 400 different types of PTMs have been discovered, including phosphorylation, acetylation, methylation, ubiquitination and SUMOylation, with phosphorylation at serine/ threonine/ tyrosine (S/T/Y) residues being the most abundant and well characterized type [5]. Different types of PTMs usually cooperate with each other to carry out specific functions. PTM at different sites of the same protein can physically interact with each other or jointly carry out a specific biological function, referred to as PTM cross-talk [6]. For example, in the human p53 protein, phosphorylation of S37 promotes phosphorylation of S33 which together activate p53’s transcriptional activity [7]. In the human CDC25C (cell division cyclin 25 homolog c) protein, phosphorylation at S214 prevents phosphorylation at its nearby site S216 and promotes cells to enter mitosis under cancerous conditions [8]. PTM cross-talks are not limited to within the same protein. For instance, ubiquitination of histone H2B forms the basis for the methylation of K79 of histone H3 [9–11]. Residue-specific cross-talk has also been shown between phosphorylation of S21 in EZH2 (enhancer of zeste homolog 2) and methylation of L27 in histone H3 [12].

Thanks to the recent advances of mass spectrometry (MS) technology, the number of known PTM sites has increased rapidly [13]. It motivated several computational studies to systematically characterize their functional relationships. Functional associations between PTM types could be revealed by statistical enrichment of different PTM type combinations observed within proteins [14], although it did not delineate relationships between individual PTM sites. At individual site level, target sites modified by more than one types of PTM is the simplest case of cross-talk [15].Apart from that, PTMs occurring in proximity were presumed to interact and used to identify motifs [16]. Indeed, phosphor-acceptor residue nearby a modified lysine (L) was found significantly more likely to be phosphorylated [17]. And the sequence and spatial distances between phosphosites are closer than expected [18]. Some disordered protein regions with very dense PTM aggregation were recently highlighted as important for combinatorial PTM regulation [15]. There was also statistical evidence supporting co-operation among locally clustered phosphorylations [19]. In addition to sequence distance, residue co-evolution between PTM sites represent another type of surrogate for their functional associations, and was used to establish a proteome-wide PTM type association network [20]. Previously, we developed a naive Bayesian classifier for PTM cross-talk prediction, which integrated sequence and structural distances, co-localization within same disordered region, as well as residue and modification co-evolution [21]. Based on a manually curated cross-talk data set, we demonstrated that integration of different features achieved better performance for cross-talk prediction than relying on individual features.

In addition, cross-talk between PTM sites can also be revealed from specialized MS experiments. For example, “top-down” or “middle-down” MS strategy can be used to directly identify PTMs co-existing within the same peptide segments [22, 23]. Quantitative MS data can also be exploited to infer co-existing PTMs based on their co-varying modification levels that change interdependently across different experimental conditions [24, 25]. However, both strategies cannot scale to the entire proteome, and were only applied to study the interactions among various modifications on histones. Another recent study investigated the co-modification of phosphorylation and ubiquitination in the entire proteome by developing a novel MS experimental strategy that enrich both modification types at the same time [26]. But it is unclear if similar methods can also be developed to study other PTM combinations. Furthermore, this strategy cannot verify if two distant co-modification exists on the same peptide sequence, and it falls short of identifying PTM cross-talks between different proteins.

Publicly available proteome-wide PTM data are mostly generated by “bottom-up” MS strategies that allow high throughput protein identification but lose connections between modifications [13, 27]. Such data are not quantitative only providing binary modification on-off status for each PTM site. Motivated by those early studies, we hypothesized that the correlation of binary modification status between two PTM sites can also suggest functional association. Following [20], functional association here is a broad concept that not only stands for cross-talk but also describes general association like involvement in the same signaling pathway or biological function. We benchmarked this idea on human phosphorylation data, because it was the only PTM type with proteome-wide coverage over large number of conditions by the time of this study. We showed that co-occurrence of phosphorylation can be used to distinguish known functionally connected phosphosite pairs from negative ones. Then we applied method to all pairwise combinations of phosphosite sites, either within proteins or between interacting partners, to identify pairs that tend to be modified under the same condition (co-occurrence). We systematically compared the observed co-occurrence with randomized data set. Site pairs showing significant co-occurring phosphorylation status were then characterized by their location preference, sharing of functional annotations and catalytic kinases, and residue co-evolution. We also compared predictions with other existing methods to highlight differences.

## Results

### Collection of condition-specific human phosphorylation data

To make use of the phosphorylation status at different conditions, we assembled human phosphorylation identified from high-throughput MS analyses across 88 different conditions from PhosphoSitePlus [28]. They include 16 human tissues as well as 28 cultured cell lines, 44 of which are disease cells (S1 Table). The conditions were selected with a minimal 1000 modification sites proteome-wide to ensure coverage. In total, the collected data contains 165,201 potential phosphosites (58.7% S, 23.6% T and 17.8% Y) of 17,819 proteins, along with their modification status across all conditions (S1 Dataset). They include 55,145 sites of 10,868 proteins (56.2% S, 18.5% T and 25.3%Y) that are phosphorylated in at least 3 different conditions (high-frequency sites). Compared with the sites phosphorylated in less than 3 conditions (low frequency sites), high-frequency sites are preferentially located in disordered protein regions (79.8% vs. 75.0%, p-value<1E-5 by permutation test), evolutionary more conserved (median Residue Conservation Score (RCS): 0.87 vs. 0.82, p-value<1E-5 by permutation test), and showed 3-fold increase of annotated functional terms (p-value<1E-5 by permutation test). The results suggest high-frequency phosphosites may be functionally more important, consistent with the previous study that suggested higher proportion of functional phosphorylations among high abundance sites [29].

### Phosphorylation sites of known functional connections are more likely to show co-occurring phosphorylation status

**Fig 1:**
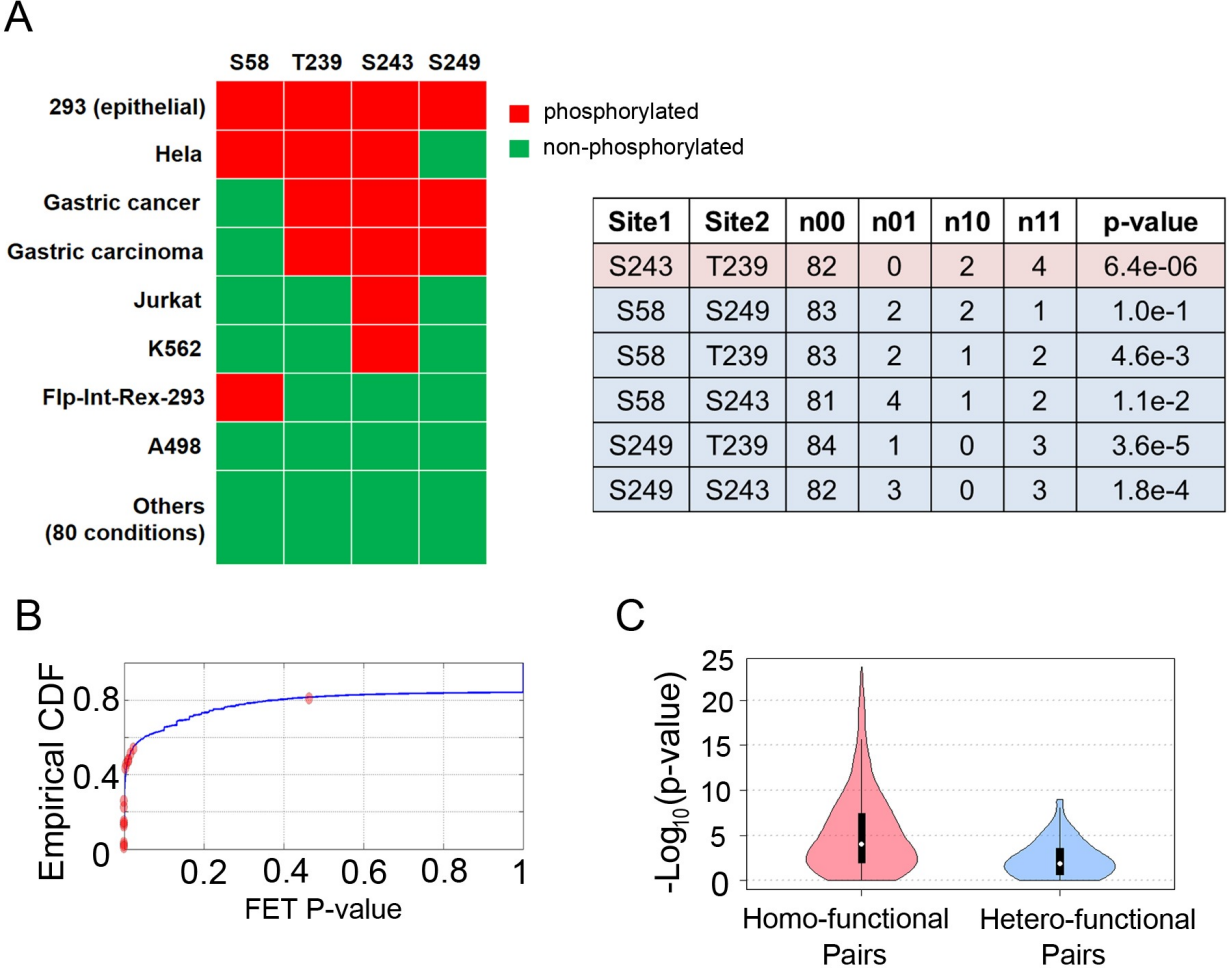
Co-occurrence of phosphorylation status can reflect known functional associations. (A) An example co-occurrence analysis on transcription factor c-Jun. There are four phosphosites (S58, T239, S243 and S249) on c-Jun. Their phosphorylation status (red: on, green: off) across 88 conditions are shown. For each pairwise combination of phosphosites, their joint phosphorylation status is summarized into a contingency table with four entries *n*_*ij*_ (*i,j* ∈ {0,1}), where *n*_*ij*_ denotes the number of times the site 1 is in state *i* site 2 is in state *j*. One-sided FET is used to test if two sites are phosphorylated together more often than expected. Consistent with the previous study that three sites (T239, S243 and S249) tend to be phosphorylated together to inhibit c-Jun’s activity in epithelial resting cells, their pairwise FET p-values are lower than their combination with S58. The most significant co-occurring pair is highlighted. (B) Cumulative distribution of co-occurrence FET p-values for 22 known cross-talk examples (red) are superimposed onto the p-values of all within-protein phosphosite pairs (blue). (C) Comparing the distribution co-occurrence FET p-values between 380 homo-functional pairs and 35 hetero-functional pairs.

To measure the tendency of two sites being phosphorylated at the same condition, we cross-tabulated the times that a pair of sites are phosphorylated across different conditions by a contingency table and calculated p-value from one-sided Fisher’s exact test (FET). Since low-frequency sites are unlikely to show statistically significant co-occurrence with other sites, we only included high-frequency sites in the co-occurrence analysis. As a known example, transcription factor c-Jun is phosphorylated at four high-frequency sites in our data (Fig 1A), three of which (T239, S243 and S249) are clustered in a short segment upstream of its DNA binding domain. They are usually phosphorylated together by GSK-3 in epithelial or fibroblast cells to inhibit c-Jun activity in resting cell states [30]. Consistent with their functional cross-talk, phosphorylation status at T239, S243 and S249 are more likely to occur together (pairwise FET p-values: 1.8E-4 to 6.4E-6) than their combinations with S58 (FET p-values: >1E-3).

As a proof of principle, we first examined the phosphosites which showed experimental validated evidence of cross-talk which were collected from literature as part of our previous study [21]. Twenty-two of them are composed of high-frequency sites in our collected data. Compared with all phosphosite pairs within proteins, their FET p-values are predominantly distributed at the lower end (median p-value: 5.0E-5; Fig 1B). We also made use of curated functional annotations for phosphosites and defined homo-functional (hetero-functional) pair as two sites that execute the same (different) biological function(s) within the same protein (Materials and Methods). The homo-functional pairs defined in this way are expected to enrich with functional associations; whereas the hetero-functional pairs are more likely functionally unrelated. Consistent with the expectation, the FET p-values of 380 homo-functional pairs are similar as known cross-talk pairs and significantly lower than those of 35 hetero-functional pairs (median: 9.74E-5 vs. 1.30E-2; Fig 1C). Since homo-functional pairs have only five overlaps with the known cross-talk set, we then combined known cross-talk and homo-functional pairs to form the positive set, and used hetero-functional pairs as the negative set (S2 Table). The co-occurrence test can be used to distinguish the two classes (Table 1), achieving an area under the ROC curve of 0.713 (S1 Fig).

**Table 1:** Comparison between known cross-talk/homo-functional pairs (positive set) and hetero-functional pairs (negative set) across different p-value threshold.

p-value cutoff	Number (%) of co-occurring pairs in the positive set	Number (%) of co-occurring pairs in the negative set	Fold increase positive/negative set
1E-6	146 (36.78%)	2 (5.71%)	6.44
1E-5	168 (42.32%)	4 (11.43%)	3.70
1E-4	198 (49.87%)	7 (20.00%)	2.49
1E-3	247 (62.22%)	10 (28.57%)	2.18
1E-2	295 (74.31%)	16 (45.71%)	1.63

The above analyses suggest that we can identify functionally associated phosphosite pairs by using the co-occurrence of their modification status across conditions.

### Proteome-wide identification of co-occurring phosphorylation pairs within proteins

We applied co-occurrence test to all pairwise combinations of high-frequency phosphosites within proteins, resulting in a total of 521,321 site pairs in 10,868 proteins. At different p-value thresholds to define co-occurring pairs, we consistently observed higher proportions among all pairs than hetero-functional pairs (Table 1), suggesting that the identified co-occurring pairs enrich true functional associations.

We also noted higher than expected proportions of co-occurrence in hetero-functional pairs. It can be explained by our incomplete knowledge of functional associations between phosphosite and/or due to confounding factors. For example, different protein abundances across conditions due to biological reasons can induce artificial co-occurrence of phosphorylation states. When a peptide segment is at high abundance level in certain conditions, its phosphorylation sites are likely to be all detected. In some other conditions, when it is at low abundance, none of the phosphorylation sites could be detected. The major effect is to inflate the significance level of association test by erroneously taken missing data as non-modification status. Although one can address this issue by incorporating protein abundance levels, this information is only available for less than 20% conditions used in the co-occurrence analysis (based on PaxDB). Furthermore, the data set we collected were generated by heterogeneous MS experiments, which employed different enrichment strategies and could introduce further variations to the digested peptide segments. The technological issue may be the major confounding factor, because we did not observe higher proportions of co-occurring pairs within housekeeping proteins [31] which are highly expressed in all tissues (S3 Table).

To evaluate the effect of confounding factors, we compared the observed proportions of co-occurring pairs in the original data under different p-value cutoffs to those of randomized data in which the number of phosphorylations at the protein level or within short peptide segments were kept the same as observed.

We first shuffled phosphorylation states among all potential phosphosites of each protein under each condition. The procedure was repeated 100 times, resulting in 135,954,681 high-frequency within-protein phosphosite pairs. We found consistent enrichments of co-occurring pairs in the original data across different p-value thresholds (Table 2). For example, at the most stringent threshold of 1E-6, 6.68% (34,835) pairs in 3,722 proteins of the original data show co-occurring phosphorylation as compared with only 0.12% (169,617) pairs in randomized data. The fold increase of co-occurring pairs in the original data decrease with relaxing p-value thresholds (Table 2).

**Table 2:** Comparison between the original and randomly permuted data in which the total number of phosphorylations at each condition kept fixed across different p-value threshold.

p-value cutoff	Number (%) of co-occurring pairs in the original data	Number (%) of co-occurring pairs in the randomized data	Fold increase Original/random data
1E-6	34,835 (6.68%)	169,617 (0.12%)	55.67
1E-5	63,760 (12.23%)	760,188 (0.56%)	21.84
1E-4	106,815 (20.49%)	2,996,345 (2.20%)	9.31
1E-3	168,250 (32.27%)	9,014,645 (6.63%)	4.87
1E-2	251,781 (48.30%)	29,953,551 (22.03%)	2.19

We next examined protein sub-sequences that range from 10 to 100 amino acids long and contain at least 5 potential phosphosites across conditions. We treated those short segments as individual proteins and generated randomized data sets as above. Again, consistent enrichment of co-occurrence can be seen in the original data. The enrichment level generally decrease with shorter fragment length (S4 Table). For example, at the fragment length of 10 and p-value cutoff 1E-5, we identified 2,357 (37.6%) co-occurring pairs compared to 147,290 (15.9%) in the randomized data. By contrast, at the length of 100 with the same p-value cutoff, the corresponding proportions in the original and randomized data are 21.3% and 2.1%. However, phosphorylation sites at proximity are known *a priori* to have functional association, so randomized data in this case are not devoid of functional association. Nevertheless, we still observed higher fold increase of co-occurrence especially at more stringent p-value thresholds (S4 Table).

Our permutation test procedures above fixed the observed number of phosphorylation sites at the protein level or within peptide segments. It can give a rough estimate of false discoveries if confounding factors mainly influences the observed number of phosphorylations, which may not be true in the process of generating real data. Despite of the caveat, we believe the observed co-occurring phosphorylation pairs can capture the functional association given the results on the known positive and negative sets. And we opted to use a stringent p-value threshold of 1E-5 to define co-occurring pairs for further functional characterization. This threshold recovers 42.3% of known cross-talk and homo-functional pairs and 11.4% of hetero-functional pairs; achieving a proper tradeoff between sensitivity and specificity.

### Functional characterization of co-occurring phosphorylation pairs within proteins

At the p-value threshold 1E-5, we identified 63,760 (12.23%) co-occurring pairs in 5,109 human proteins (S2 Dataset). For comparison, we also defined 94,391 within-protein phosphorylation pairs as controls whose FET p-values are no less than 0.5.

**Fig 2:**
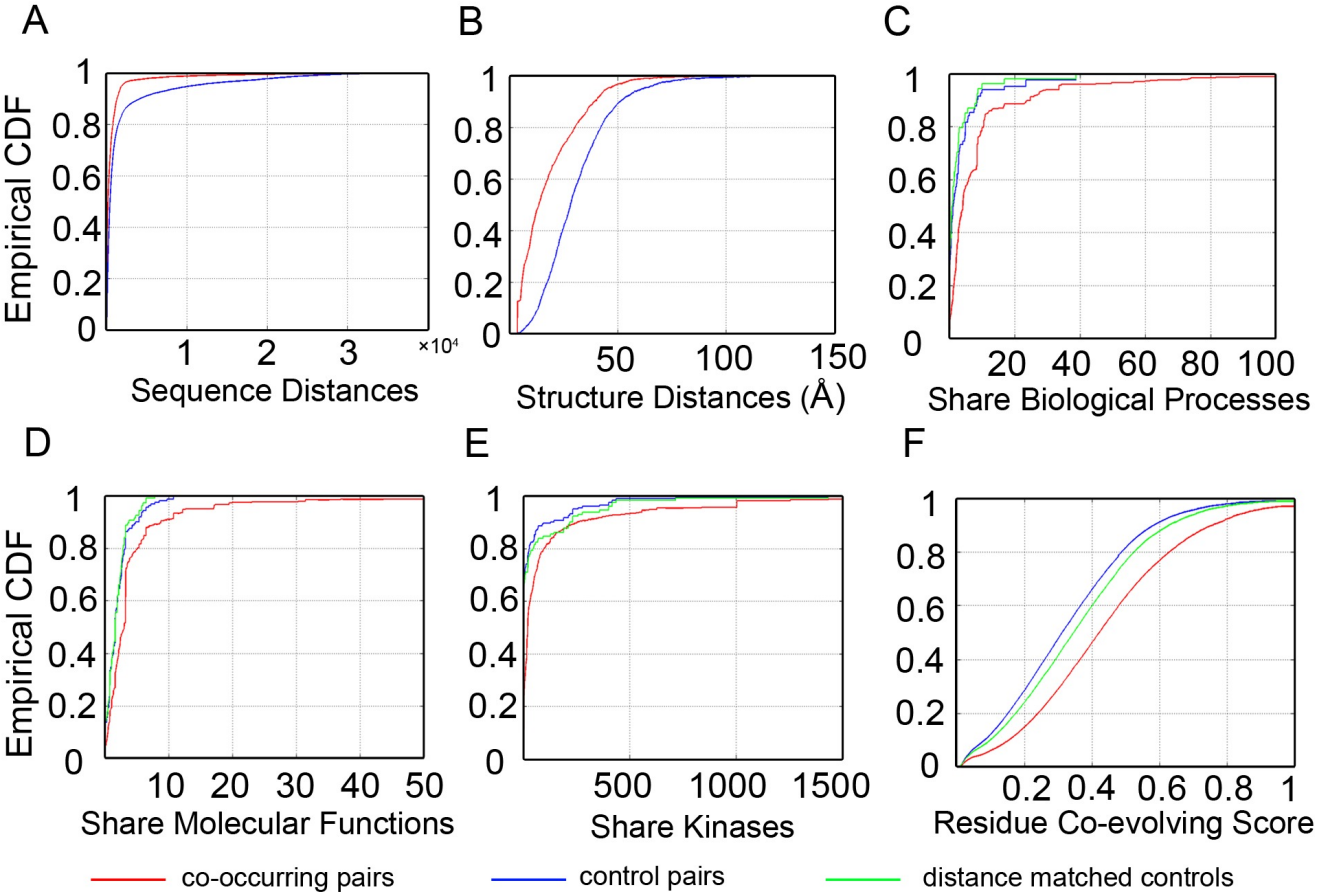
Characterization of the co-occurring phosphorylation pairs within proteins. The co-occurring and control phosphorylation pairs identified within proteins are compared on their sequence distances (A), 3D structural distances (B), scores that measure sharing annotations of biological processes (C), molecular functions (D), and catalytic kinases (E), and residue co-evolution (nMI) (F). To control for the sequence distance in comparing annotation sharing and co-evolution, co-occurring pairs were also compared with a subset of control pairs with matched distribution of sequence distances (S2 Fig).

Compared with control pairs, sites in co-occurring pairs tend to be located closer to each other in primary protein sequences (median: 166 vs. 415, p-value<1E-5 by permutation test; Fig 2A). Using the protein structure data from the PDB database [32], we were able to calculate the 3D structural distances for 1,251 co-occurring pairs and 2,022 control pairs. Phosphosites in co-occurring pairs also situated significantly closer in 3D space than control pairs (median: 13.58 Å vs. 28.21 Å, p-value<1E-5 by permutation test; Fig 2B). The results are consistent with the fact that modification sites that are closer to each other are more likely to have physical interactions and functionally associated.

We then retrieved expert curated annotations from literatures for selected phosphosites. Those include 4,971 terms of biological process for 3,133 sites, 8,434 terns of molecular function for 5,186 sites, and 364 different kinases for 7,119 sites. Compared with controls, co-occurring pairs are more likely to contain both sites with annotations (1083 vs. 256, FET p-value <1E-5). Among pairs with both sites annotated, co-occurring pairs tend to share at least one annotation (biological process: 93.80% vs. 64.62%, FET p-value = 1.2E-6; molecular function: 92.59% vs. 79.63%, FET p-value = 1.2E-4), and are more likely catalyzed by the same protein kinase (74.72% vs. 28.43%, FET p-value <1E-5). The top enriched functions among co-occurring pairs include protein degradation, induced cell growth, and cell mobility. We devised a score to measure the sharing of annotations (kinases) that accounts for the number of terms (kinases) annotated to each site and information content of each term (kinase) (Materials and Methods), and demonstrated the increased levels of function sharing among co-occurring pairs (p-values<1E-5; Fig 2C–2E). To account for the sequence distance when comparing annotation sharing, we selected a subset of control pairs with similar distribution of sequence distances as co-occurring pairs (S2 Fig), and found the difference between co-occurring and distance matched controls remained significant (p-values <1E-5; Fig 2C–2E). Together, the results suggest co-occurring pairs contains more sites of known functions, tend to be involved in the same biological pathways, and catalyzed by similar kinases, which cannot be fully explained by their physical proximity.

Because only a small proportion of phosphorylation sites had literature annotations, the functional sharing can only be analyzed for a limited number of phosphosites. To extend the functional analysis, the co-evolution of a pair of modified residues can be used as a proxy for their functional association [20]. We mapped all high-frequency phosphorylation sites to the orthologous positions across vertebrates using sequence alignments from eggNOG database [33]. Residue co-evolving scores as measured by normalized mutual information (nMI; Materials and Methods) could be calculated for 51,370 co-occurring pairs and 63,400 control pairs. Co-evolving scores of the co-occurring pairs were significantly higher than those of the control pairs (median nMI: 0.422 vs. 0.312, permutation test p-value < 1E-5; Fig 2F). Similarly, the increasing level of co-evolution in co-occurring pairs remained significant after accounting for their sequence distances (median nMI: 0.422 vs. 0.346, permutation test p-value < 1E-5; Fig 2F). We also compared evolutionary conservation of the co-occurring and the control pairs. As expected, modification sites within co-occurring pairs are slightly more conserved than control pairs (median RCS: 0.573 vs. 0.557, p-value = 2.4E-3 by permutation test; S3A Fig). The differences in conservation levels increases after excluding sites shared by the co-occurring and the control pairs (median RCS: 0.623 vs. 0.571; p-value<1E-5 by permutation test; S3B Fig).

In addition to residue co-evolution, we also tested if mouse orthologous phosphosites of human co-occurring pairs show tendency of co-occurrence of phosphorylation status. To this end, we collected 67,555 mouse phosphosites in 10,237 proteins across 34 different conditions. After removing low frequency sites (phosphorylated in less than 3 conditions), 32,887 phosphosites remained for co-occurrence analysis. We could map 23,918 (37.5%) co-occurring and 5,147 (5.45%) controls pairs from human phosphoproteins to the mouse orthologous phosphosites. Co-occurrence analysis in the mouse data shows FET p-values for mouse orthologs of the co-occurring pairs are significantly lower than that of control pairs (p-value < 1E-5 by permutation test; S4 Fig). It suggests that functional association between phosphosites captured by the phosphorylation states co-occurrence tends to be conserved from human to mouse provided their phosphorylation status are conserved.

Together, the results above lend further support to the notion that co-occurrence of phosphorylation status can be used to infer functional association between phosphosites. We note in passing that all the above results are quantitatively similar when the p-value threshold used to define co-occurring pairs was changed to 1E-4 (S5 Fig) or 1E-6 (S6 Fig).

### Identification of co-occurring phosphorylation pairs between interacting proteins

Functional associations between phosphorylation sites not only exist within same protein, but also between different proteins. Phosphorylation and other types PTMs are known to mediate the binding between PPI partners [34]. And recent studies have shown that phosphoproteins had more PPI partners than non-phosphorylated ones and both interacting proteins tend to be phosphorylated [35, 36]. It is also well characterized in signal transduction pathways that phosphorylation activates kinases to phosphorylate their substrates (kinase cascade). Some stable kinase-substrate relationships can also be captured by PPI. So we extend the co-occurrence analysis to the phosphosites between interacting proteins. We used 13,944 experimental validated high quality PPI pairs from CCSB Human Binary Interactome database [37].

A total of 55,145 high-frequency sites could be mapped to the CCSB PPI set, resulting in 132,360 pairs between 2,959 interacting partners. One-sided FET was performed to test the tendency of co-occurrence of modification status between each phosphosite pairs.

**Fig 3:**
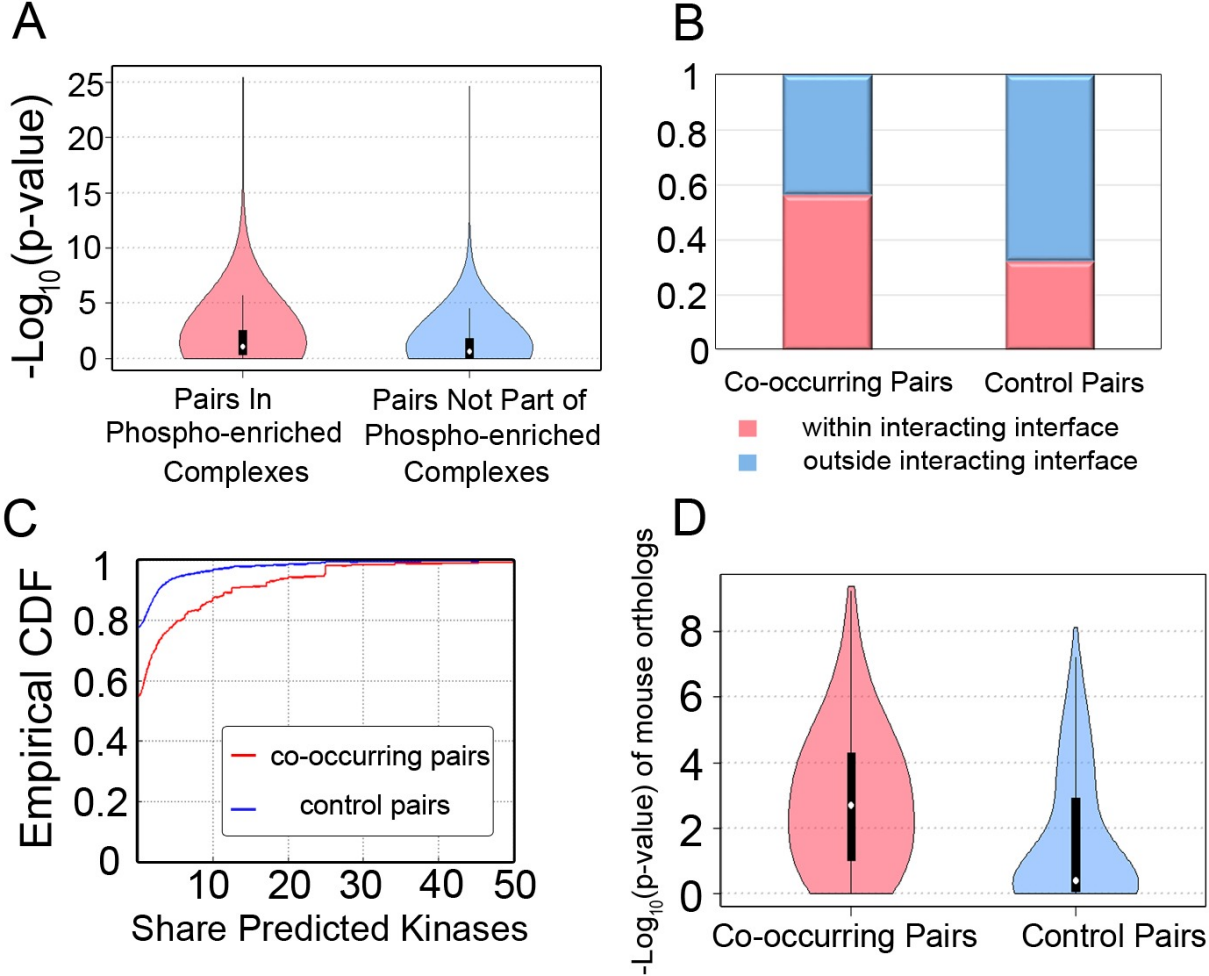
Characterization of co-occurring phosphorylation pairs between interacting proteins in the CCSB PPI set. (A) Co-occurring pairs located in phosphorylation enriched protein complexes [15] are enriched with small FET p-values. Compared with control pairs, co-occurring pairs are more likely to co-localize in the PPI interfaces (B), and be catalyzed by the same predicted kinases (C). (D) The mouse orthologous phosphosites of human co-occurring pairs also show the tendency of being modified under same conditions.

As within-protein analyses, we first contrast phosphosite pairs that have higher chances of functional associations with those most likely unrelated. One recent computational study found that phosphorylations are selectively accumulated in protein complexes, and some complexes tend to integrate phosphorylation signals on distinct subunits [15]. So we examined the co-occurrence between phosphorylations from different sub-units of phosphorylation enriched complexes reported by that study. A total of 3,654 phosphosite pairs can be mapped to 30 protein pairs within same complexes (positive set); and 124,594 phosphosite pairs mapped to 4,719 protein pairs that are not part of the complexes (negative set). Phosphosite pairs in the positive set are enriched with small FET p-values as compared with the negative set (Fig 3A, Table 3). It is consistent with the functional associations between phosphosites within some of these complexes.

**Table 3:** Comparison between the positive and negative sets between interacting proteins across different p-value thresholds. The positive set is defined as phosphosite pairs in which both sites are located within phosphorylation enriched protein complexes [15]. The negative set is defined as phosphosite pairs in which at least one site cannot be mapped to those complexes.

p-value cutoff	Number (%) of co-occurring pairs in the positive set	Number (%) of co-occurring pairs in the negative set	Fold increase Positive/negative set
1E-7	93 (2.55%)	444 (0.36%)	7.14
1E-6	163 (4.46%)	1,359 (1.09%)	4.09
1E-5	262 (7.17%)	3,230 (2.59%)	2.77
1E-4	422 (11.48%)	7,171 (5.76%)	2.00
1E-3	699 (19.13%)	14,052 (11.28%)	1.69

Phosphorylation states co-occurrences between interacting proteins may also be confounded by the co-varying abundance level of interacting proteins or their digested peptides across conditions. Using the same permutation approach in which the total number of phosphosites of each protein was kept fixed, we also observed a significant excess of observed co-occurring pairs compared with randomized data, with decreasing level of fold increase at relaxing p-value thresholds (Table 4). We also examined short peptide fragments (10–100 amino acids) containing at least 5 potential phosphosites. By controlling the observed number of phosphorylations within each short segment, we can observe a higher proportion of co-occurring pairs from short segments between interacting proteins than random expectations (S5 Table). The same caveat as in the within-protein analysis applies to interpret the result of randomization.

**Table 4:** Comparison between the original and randomly permuted data in which the total number of phosphorylations of each protein of the interacting pair at each condition keep fixed.

p-value cutoff	Number (%) of co-occurring pairs in the original data	Number (%) of co-occurring pairs in the randomized data	Fold increase Original/random data
1E-7	556 (0.42%)	1,434 (0.003%)	132.49
1E-6	1,575 (1.19%)	12,367 (0.027%)	44.07
1E-5	3,610 (2.73%)	81,611 (0.18%)	15.17
1E-4	7,809 (5.90%)	415,653 (0.92%)	6.41
1E-3	15,231 (11.51%)	1,420,678 (3.14%)	3.67

Compared to within-protein analysis at the same p-value threshold, the observed proportion of co-occurring pairs between interacting proteins is much lower. For example, at the p-value cutoff 1E-5, 2.73% of all high-frequency phosphosite pairs (and 7.17% of those within same phosphorylation enriched complexes) were identified as co-occurring pairs, whereas 12.23% were found within proteins. Notably, the proportions of identified co-occurring pairs among all pairs are consistently higher than those of the negative set (Table 3), although phosphosite pairs in the negative set defined above are not fully unrelated in functions, suggesting that co-occurrence analysis can still uncover some functional associations.

### Functional characterization of co-occurring phosphorylation pairs between interacting proteins

For further functional characterization, we defined co-occurring phosphorylation pairs between interacting proteins by FET p-value less than 1E-5 and control pairs by p-value no less than 0.5. A total of 3,610 co-occurring pairs (S3 Dataset) and 53,016 control pairs are identified and used in the following comparisons.

To evaluate if two phosphosites of co-occurring pairs tend to be closer in space, we test whether two sites of co-occurring pairs are more likely to co-localize at the interaction interfaces of interacting proteins. Structurally resolved interactions for 6,585 human protein pairs were obtained from the INstruct database [38]. A total of 427 co-occurring pairs are found between protein pairs with interaction domain information, and 56.44% of them are in the interacting interfaces. By contrast, among 1872 control pairs mapped to Instruct, only 32.32% of them are in the interfaces (Fig 3B).

To test if co-occurring pairs tend to be catalyzed by similar kinases, we used a computational approach (Materials and Methods) to link phosphosites to 39 different kinases. In total, kinase information could be obtained for both sites of 882 co-occurring pairs and 6,446 control pairs. Co-occurring pairs are more likely to share at least one kinase (34.92% vs. 14.71%, p-value<1E-5 by FET) and have higher kinase sharing scores (Fig 3C). Together, the above results can be interpreted that physically binding proteins tend to be phosphorylated by the same enzyme, and their structural proximity would facilitate this process.

We also analyzed residue co-evolution and conservation of co-occurrence in mouse proteome. For phosphosite pairs whose co-evolving score can be calculated (54,46% co-occurring pairs and 57.88% control pairs), the co-occurring pairs have slightly higher nMI scores than controls (nMI: 0.2 vs. 0.19, p-value = 0.019). For phosphosites whose phosphorylation status are conserved in the mouse orthologous positions, they contain 625 co-occurring and 1,442 control pairs. FET p-values of the co-occurrence test in mouse proteome were significantly lower for the orthologs of co-occurring pairs than control pairs (median: 0.00193 vs. 0.409, p-value<1e-5 by permutation test; Fig 3D). The results resemble those of within-protein pairs showing co-occurring phosphosite pairs are more likely to co-evolve and their co-occurrence states are conserved in mouse.

### Comparison with PTMcode

The PTMcode database (http://ptmcode.embl.de) contains PTM associations of different PTM types collected based on multiple evidence channels. Among phosphosites, their functional relationships were computationally predicted based on residue co-evolution and space proximity in 3D structure [39], with the overwhelming majority (>99%) based on co-evolution.

To compare with PTMcode, we focus on the common set of 4,617 proteins on which the functional relationship between phosphosites can be analyzed by both PTMcode and our study. On these genes, 504,849 phosphosite pairs were annotated by PTMcode as functionally associated, and 30,779 phosphosite pairs were identified as co-occurring pairs (defined by FET p-value <1E-5). The much higher number of pairs identified by PTMcode mainly because co-occurrence analysis is limited to high-frequency phosphosites. Indeed, only 62,732(12.43%) of pairs identified by PTMcode are composed of both high-frequency sites. Intersecting PTMcode and co-occurring pairs results in an overlap of only 9,809 pairs.

We further compared 495,040 pairs specific to PTMcode with 20,970 pairs specific to co-occurrence analysis. Co-occurrence specific predictions contain higher proportion of pairs with both functional annotated sites than PTMcode specific predictions (313 (1.49%) vs. 2,378 (0.48%), p-value<1E-5 by FET), and higher proportion of pairs with kinase information (334 (1.59%) vs. 4,478 (0.90%), p-value<1E-5 by FET). Among pairs with functional annotations, co-occurrence specific pairs more likely to share functional annotations (286 (91.37%) vs. 1,736 (73.00%), p-value<1E-5) and at least one catalytic kinase (263 (78.74%) vs. 2,761 (61.66%), p-value<1E-5), and have slightly higher functional sharing scores (S7A–S7C Fig).

PTMcode v2 also predicted functional associations for PTM sites between high-confidence interacting proteins annotated by the STRING database [40], which comprehensively catalogs known and predicted interacting proteins. Given the difference of PPI sets and data requirement for different methods, comparison between them is difficult. Here we focus on the 102 protein pairs that were analyzed by both PTMcode and by our study. On these protein pairs, 9,177 were annotated as functionally associated by PTMcode, 219 were identified as co-occurring pairs, with only 68 pairs in common. Bioinformatics analysis predicted kinases for 31 (14.16%) of co-occurrence specific pairs and 214 (2.33%) PTMcode specific pairs. Co-occurrence specific pairs are more likely to be catalyzed by the same predicted kinase (11 (35.48%) vs. 46 (21.50%)), and have higher kinase sharing scores (S7D Fig).

The results indicated that although the application of co-occurrence is limited by the availability of the data which will continue to expand in the future, it can be complementary to the existing method like residue co-evolution in predicting functionally associated phosphorylation. And co-occurrence analysis is more likely to identify site pairs with share functional annotations and catalyzed by the same kinase.

## Discussion

More than half of all human proteins can be phosphorylated. And phosphorylation dynamically regulates enzymatic activity, protein stability, subcellular localization, and transmit signals to downstream pathways, etc. [41]. Its function can be fine-tuned by multiple phosphorylation sites within protein or protein complex [42]. To better understand function relationships between different phosphosites, in this study, we exploited co-occurrence of phosphorylation status across conditions from public available high-throughput MS data.

To mitigate the influence of confounding factors, we opted to use a stringent p-value threshold in statistical test. And a series of simulations were performed to systematically compared the number of identified co-occurring phosphosite pairs with random expectation controlling for the number of phosphorylations within protein or short peptide segments. Although we consistently observed higher proportion of co-occurring pairs than random permutation, we did not derive false discovery rates from this comparison because randomization may not fully capture the effect of confounding factors and would most likely under-estimate false positives. In the future, incorporating protein abundance information and applying uniform MS protocols across conditions can better address this issue. We also benchmarked the discriminative performance of the co-occurrence p-value in classifying the positive set that are enriched for known functional associations and the negative set that are more likely to be functionally unrelated. The performance based on this type of analysis should be interpreted as a lower bound, given the lack of golden standard for the positive and negative sets. Taken together the method’s ability to distinguish the positive and negative sets, higher discovery rates in the original than randomized data, and high *a priori* functional association between phosphosites within proteins, we believe the identified co-occurring pairs within proteins are enriched for functional associations.

We then sought additional evidence to support the functional relevance of the identified co-occurring pairs by their sequence/structural proximity and residue co-evolution, which are also two commonly used measures of functional association [16, 20]. Phosphorylations closer to each other have higher chance of physical interaction. Indeed, for more than two thirds of known cross-talk pairs, two phosphosites are within 20 amino acids. Common mechanisms of functional association include that phosphorylation at one site facilitate the phosphorylation the other site (e.g., S33 and S37 of p53 [7]), or several phosphosites need to be simultaneously phosphorylated to fulfill a molecular function (e.g., T239, S243, S249 of c-Jun [30], Y342 and Y346 of Sky [43]). Functional associations are certainly not limited to nearby phosphosites. Among 63,760 significant co-occurring pairs within proteins, the primary sequence distance is less than 20 amino acids for 26.77% of all pairs, and more than 57.63% of them are separated by at least 100 amino acids. A similar proportion (60.27%) were also observed for functionally associated phosphosite pairs in PTMcode [39] identified by the co-evolution method. Our comparison with PTMcode showed limited overlap and suggested co-occurrence analysis can be used as a complement. Identified co-occurring pairs include several well characterized long-range functional cross-talks, including Y707 and Y806 of CDCP1 that are phosphorylated by Src family kinases (SFKs) and activate CDCP1 to promote cell growth and SFK activities [44], S22 and S390 of lamin-A that are phosphorylated by BGLF4 and promote the reorganization of the nuclear lamina [45], and S612 and T365 of Rb protein which function together to prevent its association with E2F transcription factor [46]. Notably, the first two examples were not uncovered by PTMcode.

While it is plausible that those long range functional cross-talks play roles in allosteric and orthosteric regulation of proteins [47], another possibility could be that a group of phosphosites in a protein region contributes to the modification collectively through an aggregate property irrespective of precise locations [48, 49], e.g. through bulk electrostatics [50]. Under this model, natural selection would act to maintain the total number of phosphosites but individual phosphosites may not be conserved [51]. Co-evolution may fail to identify the functional association in such cases. A well-studied example is pre-replication complex: several sub-units in human (CDC6, CDT1, MCM2, MCM4, and ORC1) contain cluster of phosphosites that are phosphorylated by CDKs. Those phosphosites showed rapid evolutionary turnover even when the local cluster of site is preserved. Co-occurrence analysis identified functional associated pairs in all five proteins, notably no evidence of co-evolution for phosphosites of ORC1 and CDT1. Other examples of clustered co-occurring phosphorylations include DNA repair protein ERCC5, DNA polymerase subunit RFC1, RNA polymerase subunit POLR2A, etc.

Throughout this manuscript, we focus on co-occurrence or positive correlation of modification status, because we found few negative correlations in the original and randomized data (S6 Table). Although most known cross-talks between phosphorylations are positive (activating or co-operative), negative (inhibit or steric hindrance) example does exist, for example the mutual inhibitory between phosphorylation of S214 and S216 of CDC25C [8]. In other cases, phosphorylations at different sites in the same protein can have opposite effects on protein activity causing activation or inhibition of downstream function [52]. We examined the modification status for the CDC25C example and 35 hetero-functional pairs in which one site has activating function and the other has inhibiting function (S7 Table), but found none of them show negative correlation trend. There are two possible reasons. First, the steric hindrance case cannot be revealed in the data because we observe both sites are phosphorylated under multiple conditions. In the case of CDC25C, we indeed observed moderate evidence of co-occurrence between S214 and S216 (p-value = 1.1E-4). It is because the phosphorylation status detected by the “bottom-up” MS technology reflects the stoichiometry of phosphorylation in a pool of multiple isoforms, even if two sites are mutually exclusive on one isoform, they can still be detected under the same condition because both isoforms exist. This issue can be address by “top-down” or quantitative MS approaches. Second, sites with opposite functional categories cannot also be identified as negative correlation because in many conditions neither site is phosphorylated due to biological (low protein abundance) or technical reasons (low enrichment of digested peptides). Indeed, over 80% of phosphosites analyzed in this study are phosphorylated in less than 10 conditions and over 60% are phosphorylated in less than 5 conditions (S8 Fig). After removing conditions when neither sites are phosphorylated, we found that hetero-functional pairs tend to be phosphorylated in only one site, consistent with their opposite functions. The two reasons above can be unified under the same statistical principle that the presumed negative correlation is indeed conditional association. For mutual inhibitory pairs, negative correlation is conditional on being on the same protein isoform; for hetero-functional pairs, negative correlation is conditional on at least one site is phosphorylated. The co-occurrence analysis of binary phosphorylation status can only identify marginal association which is not necessarily the same as conditional association.

We also applied co-occurrence analysis to interacting protein pairs. Given lower *a priori* of functional association for sites between proteins and issues of confounding, we expect to observe higher rate of false positives. Despite of this, the observed global trends support the co-phosphorylation of interacting proteins by the same kinase [35, 53], and the role of signal integration in phosphorylation enriched protein complex [15]. We found several between co-occurring pairs with suggestive functional evidence. For example, GRB2 and one of its interaction partner GAB2 contain more than 20 inter-protein co-occurring phosphosite pairs. GRB2 is an adaptor protein involved in signal transduction and cell communication [54, 55], and GAB2 is a multi-site docking protein and serve as the gateway into GRB2 activation [56]. The co-occurring phosphosite pairs between these two proteins may play roles in mediating interaction between different signaling pathway and signal integration. As another example, Y705 of STAT3 and Y323/Y352 of SYK show co-occurrence under more than half of biological conditions consistent with the established genetic and biomedical evidence that STAT3 is a substrate of SYK [57], so the co-occurrence in this case may represent part of signal cascading. In this study, interacting proteins are defined by a set of experimentally validated high-quality PPI. One limitation is that PPI may not include many kinase-substrate relationships [58]. We collected 8,666 known kinase-substrate pairs from PhosphoSitePlus and only found 10 overlap with the CCSB PPI, possibility because kinase usually interact with phosphosites in a transient manner. When applying the co-occurrence analysis to kinase-substrate pairs, the identified proportion of co-occurring pairs are similar as the PPI set (S8 Table). The enrichment of co-occurring pairs in phosphorylation enriched complexes and in interaction interfaces are also observed (S9 Fig). For the co-occurrence analysis between interacting proteins, we suggest it be applied to the cases in which there is strong evidence for functional association between phosphorylations, and interpretation should be made by considering the function of interacting proteins.

Together our study shows that co-occurring phosphorylation are functionally associated, and suggests the utility of mining co-occurrence of modification status to reveal functional association between PTM sites. With the increasing coverage of other PTM types, the co-occurrence can potentially be integrated with other methods to identify novel functional associations between different PTMs. We also found that phosphosites of the co-occurring pairs are more likely to contain functional annotations and evolutionary conserved, suggesting they are more likely to be functional. While previous studies to prioritization functional phosphorylations focus on individual sites [17, 29, 59], the functional associations identified by the co-occurrence analysis in our study can provide further lines of evidence for this purpose.

## Materials and methods

### Compilation of phosphorylation sites under different conditions

We downloaded all experimentally observed human phosphorylation sites from the PhosphoSitePlus database (http://www.phosphosite.org, last access: 2016–02) [28]. The observed modification sites were further stratified into different laboratory (cell line vs. tissue) and physiological (disease vs. non-disease) conditions, resulting in a total 656 data files. To ensure proteome-wide coverage, we only retained 88 different conditions with at least 1000 modification sites. The final data set used in the analysis contains 55,145 sites of 10,868 proteins and their binary phosphorylation status under the 88 conditions (S1 Table).

To investigate if the observed functional association between phosphorylation sites in the human proteome were conserved in mouse, we also downloaded all mouse phosphorylation sites and processed with the same criteria as human. The final data set includes 67,555 sites of 10,237 mouse proteins along with their phosphorylation status under 34 different conditions. Orthologous proteins of human and mouse were downloaded from InParanoid database v8 [60]. Only the 1-to-1 orthologs with confidence scores greater than 0.9 were kept. To map human phosphorylation sites to the orthologous positions in mouse, human and mouse protein sequences from the UniProt database were aligned by MUSCLE 3.8.31 [61].

We mapped phosphosites to the disordered protein regions predicted by DisEMBL [62]. The 3D structural positions were obtained from PDB database [32]. And structural distance between phosphosites was defined as the distance between the two α-carbon atoms adjacent to the carboxyl group of amino acids.

### Identification of co-occurring phosphosites pairs

For each pair of phosphorylation sites, we cross-tabulated the times that two residues are phosphorylated under different conditions into a 2-by-2 contingency table. The p-value of one-sided FET was used to evaluate the tendency of phosphorylation to co-occur under the same conditions. The procedure applied to phosphosites within proteins or between pre-specified protein pairs. In the main text, we chose the threshold of 1E-5 to define co-occurring pairs. Residue sites that are phosphorylated in less than 3 conditions were removed prior to the calculation because pairs with one rarely phosphorylated site cannot not reach the desired significance level.

To explore how many co-occurring phosphorylation pairs can be identified in the randomized data sets, we performed a series of permutation tests in the following way. For each protein, we first identified its potential phosphosites which were modified at least once across conditions. Then we randomly introduced the same amount of phosphorylation status as observed at each condition among those potential sites. The process was repeated 100 times; and one-sided FET was performed as above to identify co-occurring pairs within proteins or between protein pairs. The above procedure generated randomized data sets by fixing the total number of modification sites within protein, we also considered controlling the number modification sites within a short peptide segment. To do this, we identified all non-overlapping windows of 10, 20, 50, 70 or 100 amino acids long which contain no less than 5 potential phosphorylation sites, treated them as individual proteins and then applied the same permutation procedure as above to generate randomized data.

### Co-evolving and conservation scores

We downloaded multiple sequence alignment (MSA) and the species tree of vertebrates non-supervised orthologous groups (veNOG) from the eggNOG database (v4.5) [33]. Human phosphorylation sites were mapped to the orthologous positions across vertebrates. The normalized mutual information (nMI) [63–65] was used to measure the co-evolution of residues at two modification sites:

$$nMI(X;Y)=\frac{MI(X;Y)}{\sqrt{\sum_{x\in A_{X}}p(x)\log(p(x))\sum_{y\in A_{Y}}p(y)\log(p(y))}}$$ (1)

$$MI(X;Y)=\sum_{y\in A_{Y}}\sum_{x\in A_{X}}p(x,y)\log(\frac{p(x,y)}{p(x)p(y)})$$ (2)

where x and y represent amino acids or alignment gaps at the orthologous positions of human phosphosites *X* and *Y* across species; *p* (*x*) and *p* (*y*) are the marginal frequencies, and *p* (*x*, *y*) is the joint frequency of *x* and *y* across MSA. Only sites with at least three non-conserved residues across species were included in the nMI calculation.

Residue conservation score (RCS) was used to measure the conservation of phosphorylation sites, and was calculated using the method of [20]. Briefly, for each site we first determine the maximum branch length (MBL) in the species based on residues the species that have the same amino acid as human. MBL is calculated as the ratio relative to the two most distant species. Then we built a sub-tree containing the most common ancestor of species with the same amino acid as human, and calculated the ratio of conserved residues (RCR) among species in the sub-tree. Finally, RCS was obtained by the product of MBL and RCR. Given both MBL and RCR are defined as ratios, RCS will take values from 0 to 1.

### Functional annotations of phosphosites

We retrieved experts curated annotations about biological process, molecular function, and catalyzing kinases for phosphosites from the PhosphoSitePlus database. For sites with annotations, they were classified into three board functional categories (activate/ inhibit/ dual). We defined homo-functional pair as two phosphosites that belong to the same category and share at least one annotation term, and hetero-functional pair as those that belong to different categories and do not share any annotation term.

To measure the sharing of functional annotations between two phosphorylation site, we devised a score that account for the information content (specificity) of annotation terms which is defined as:

$$\frac{1}{N_{A}\times N_{B}}\sum_{A_{\text{i}}\in A,B_{j}\in B}\left[I\{A_{i}==B_{j}\}\times \frac{1}{F(A_{i})}\right]$$ (3)

where *A* and *B* denote the sets of annotation terms for two modification sites, *N*_*A*_ and *N*_*B*_ are the cardinality of the sets, and *A*_*i*_ and *B*_*j*_ are individual terms. *F*(*A*_*i*_) is the frequency of the term *A*_*i*_ that appear in the entire database; *I*{*A*_*i*_ == *B*_*j*_} is an indicator function taking value of 1 only if *A*_*i*_ and *B*_*j*_ are the same. Effectively, this formula accounts for the number of annotations of each site and puts higher weights to the sharing of specific (low frequency) terms than the general (high frequency) terms.

### Predicting the catalytic kinases of phosphorylated sites

We used position weight matrix (PWM) to represent kinase-specific substrate motifs defined as the 15 amino acids sequence context centering on the modification site:

$$M=\left[\begin{array}{c} M_{A,1},M_{A,2},\ldots ,M_{A,14},M_{A,15}\\ M_{C,1},M_{C,2},\ldots ,M_{C,14},M_{C,15}\\ M_{D,1},M_{D,2},\ldots ,M_{D,14},M_{D,15}\\ \ldots \\ M_{Y,1},M_{Y,2},\ldots ,M_{Y,14},M_{Y,15} \end{array}\right]$$ (4)

$$M_{k,j}={log}_{2}\left(\frac{q_{k,j}}{b_{k,j}}\right)$$ (5)

where *k* ∈ {A,C,D,E,F,G,H,I,K,L,M,N,P,Q,R,S,T,V,W,Y} *i,j* ∈ {1,2,…,15}. *q*_*k,j*_ and *b*_*k,j*_ denotes the frequency of amino acid *k* at motif position *j* in the foreground and background set respectively.

To derived the foreground set, we downloaded all known kinases-substrate relationships from the PhosphoSitePlus database, and selected 39 kinases with at least 50 different site-specific substrates. Then for each kinase, the sequences of 15 residues centering on the substrate phosphorylated sites were extracted and positional frequencies of amino acids were calculated. For the background set, we retrieved 15 amino acid context centering on S, T, or Y sites of all human proteins.

To predict whether one phosphosite was catalyzed by a kinase, we made use of this kinase’s PWM and score the phosphosite given its sequence context:

$$Score=\sum_{j=1}^{15}M_{s_{j},j}$$

where *s*_*j*_ is the amino acid at motif position *j* for this phosphosite. For each kinase, we first scored all its known substrates, and took the median score as the cutoff for prediction. Then for phosphorylation sites with unknown kinase, if its score by the kinase’s PWM exceeded the cutoff, the site was predicted to be catalyzed by this kinase. Note, in this way one phosphosite can be predicted to be catalyzed by multiple kinases.

## Supporting information

S1 Dataset Human phosphosites and their phosphorylation status under all 88 laboratory or physiological conditions (Table S1). The data file is essentially a 2-D matrix. Each row denotes a phosphosite; and each column represents one condition. The on-off modification status is shown as 0s and 1s.
(TXT)

S2 Dataset Phosphosite pairs within proteins that show co-occurring phosphorylation status. The data file contains co-occurring phosphosite pairs identified by FET p-value <1E-4. For each pair, its contingency table and one-side FET p-value is shown.
(TXT)

S3 Dataset Phosphosite pairs between interacting proteins that show co-occurring phosphorylation status. The CCSB PPI database is used to define interacting proteins. The data file contains co-occurring phosphosite pairs identified by FET p-value <1E-4 in the union of two sets. For each pair, its contingency table and one-side FET p-value is shown.
(TXT)

S1 Table The eighty-eight laboratory or physiological conditions. For each condition, it shows a brief description, total number of phosphorylations, and literature references.
(XLSX)

S2 Table Application of co-occurrence test to the known positive and negative sets. The positive set is comprised of 397 known cross-talk or homo-functional pairs; the negative set is comprised of 35 hetero-functional pairs. For each pair of phosphosite, it shows the contingency table summarizing their joint modification status and one-sided FET p-value.
(XLSX)

S3 Table Number of observed co-occurring pairs identified within proteins of housekeeping genes. It compares, across different p-value thresholds, the number and proportion of co-occurring pairs within proteins encoded by housekeeping genes and all others. Housekeeping genes are defined by an updated list of 3804 genes [31].
(DOCX)

S4 Table Number of co-occurring pairs within same short peptides across different p-value thresholds (related to Table 2). It compares the proportion of co-occurring pairs identified within short peptides (10–100 amino acids) that contain at least 5 phosphosites and randomly permuted data where the number of phosphorylations of each peptide at each condition is fixed.
(XLSX)

S5 Table Number of co-occurring pairs between short peptides of interacting proteins across different p-value thresholds (related to Table 4). It compares the proportion of co-occurring pairs identified between short peptides (10–100 amino acids) of interacting proteins which contain at least 5 phosphosites and randomly permuted data where the number of phosphorylations of each peptide at each condition is fixed.
(XLSX)

S6 Table Number of negatively correlated pairs. One-sided FET is applied on both original data randomized data to identify negative correlated pairs. The results are shown across different p-value thresholds.
(DOCX)

S7 Table Example of phosphosite pairs of presumed “negative correlation”. The list includes one example of known cross-talk where phosphorylation at one site inhibit the phosphorylation at the other (CDC25). It also includes 35 hetero-functional pairs within proteins with either site carrying out distinct functions under different conditions.
(XLSX)

S8 Table Number of co-occurring pairs between known kinases and substrates across different p-value thresholds. A total of 18,771 high-frequency phosphosites were mapped to known kinase-substrate pairs resulting in 503,077 phosphosite pairs between 284 kinases and 1,797 substrates. (A) Comparison between the positive and negative sets. The positive set is defined as phosphosite pairs in which both sites are located within phosphorylation enriched protein complexes [15]. The negative set is defined as phosphosite pairs in which at least one site cannot be mapped to those complexes. (B) Comparison between the original and randomly permuted data in which the total number of phosphorylations of each protein of the interacting pair at each condition keep fixed.
(DOCX)

S1 Fig The ROC curve for classifying known cross-talk/homo-functional pairs and hetero-functional pairs based their co-occurrence of phosphorylation status. It shows true positives (sensitivity) and false positives (1-specificity) with respect to different FET p-value thresholds.
(TIF)

S2 Fig The distribution of sequence distances for the co-occurring pairs (A) and matched control pairs (B) within proteins.
(TIF)

S3 Fig Comparison of the evolutionary conservation levels between phosphosites in the co-occurring pairs and the control pairs. Conservation level is measured by residual conservation score. Comparisons are made using either all phosphosites (A), or after removing sites that are shared by the co-occurring and the control pairs (B).
(TIF)

S4 Fig FET p-values of mouse phosphosite pairs orthologous to human. The mouse orthologous phosphosites of human co-occurring pairs are more likely to be modified under the same conditions than those of control pairs.
(TIF)

S5 Fig Characterization of the co-occurring phosphorylation pairs within proteins (related to Fig 2). The co-occurring pairs within proteins are defined by FET p-value<1E-4, and controls p-value> = 0.5. The co-occurring and control pairs are compared on their sequence distances (A), 3D structural distances (B), scores that measure sharing annotations of biological processes (C), molecular functions (D), and catalytic kinases (E), and residue co-evolution (nMI) (F). To control for the sequence distance in comparing annotation sharing and co-evolution, co-occurring pairs were also compared with a subset of control pairs with matched distribution of sequence distances. The co-occurring pairs defined by FET p-value<1E-5 are also superimposed on the plots.
(TIF)

S6 Fig Characterization of the co-occurring phosphorylation pairs defined by FET p-value<1E-6 within proteins. The co-occurring pairs within proteins are defined by FET p-value<1E-6, and controls p-value> = 0.5. The co-occurring and control pairs are compared on their sequence distances (A), 3D structural distances (B), scores that measure sharing annotations of biological processes (C), molecular functions (D), and catalytic kinases (E), and residue co-evolution (nMI) (F). To control for the sequence distance in comparing annotation sharing and co-evolution, co-occurring pairs were also compared with a subset of control pairs with matched distribution of sequence distances. The co-occurring pairs defined by FET p-value<1E-5 are also superimposed on the plots.
(TIF)

S7 Fig Comparison with PTMcode. For phosphosite pairs within proteins, pairs that identified only by PTMcode and only from co-occurrence analysis are compared by the scores that measure sharing annotations of molecular functions (A), biological processes (B), and catalytic kinases (C). For phosphosite pairs between interacting proteins, PTMcode specific and co-occurrence specific pairs are compared by the score that measures sharing of computationally predicted kinases (D).
(TIF)

S8 Fig Distribution of frequencies that phosphosites used in the co-occurrence analysis are modified under 88 conditions. (TIF)

S9 Fig The enrichment of kinase-substrate co-occurring pairs in phosphorylation enriched complexes (A) and in interaction interfaces (B).
(TIF)
