# Supplementary material for: Co-occurring protein phosphorylation are functionally associated
Source: PLoS Comput Biol. 2017 May 1;13(5):e1005502. doi: 10.1371/journal.pcbi.1005502 (PMC5432191; doi:10.1371/journal.pcbi.1005502)
Supplement: S3 Table — It compares, across different p-value thresholds, the number and proportion of co-occurring pairs within proteins encoded by housekeeping genes and all others. Housekeeping genes are defined by an updated list of 3804 genes [31]. (DOCX) [file pcbi.1005502.s006.docx]

**Number of co-occurring pairs in housekeeping genes vs all other genes.**

| **p-value cutoff** | **Number (proportion) of co-occurring pairs in housekeeping genes** | **Number (proportion) of co-occurring pairs in other genes** |
| --- | --- | --- |
| 1e-6 | 7,986 (6.87%) | 34,835 (6.68%) |
| 1e-5 | 13,636 (11.73%) | 63,760 (12.23%) |
| 1e-4 | 22,836 (19.65%) | 106,815 (20.49%) |
| 1e-3 | 36,130 (31.09%) | 168,250 (32.27%) |
| 1e-2 | 55,786 (48.00%) | 251,781 (48.30%) |
