# Supplementary material for: Co-occurring protein phosphorylation are functionally associated
Source: PLoS Comput Biol. 2017 May 1;13(5):e1005502. doi: 10.1371/journal.pcbi.1005502 (PMC5432191; doi:10.1371/journal.pcbi.1005502)
Supplement: S6 Table — One-sided FET is applied on both original data randomized data to identify negative correlated pairs. The results are shown across different p-value thresholds. (DOCX) [file pcbi.1005502.s009.docx]

**Number of negatively correlated pairs.**

(A) Within same proteins

| **p-value cutoff** | **Number (proportion) of negative pairs in the original data** | **Number (proportion) of negative pairs in the randomized data** |
| --- | --- | --- |
| 1e-6 | 0 | 0 |
| 1e-5 | 0 | 0 |
| 1e-4 | 0 | 0 |
| 1e-3 | 4 (7.67e-6) | 22 (1.61e-9) |
| 1e-2 | 28 (5.37e-5) | 453 (3.32e-6) |

(B) Between interacting proteins

| **p-value cutoff** | **Number (proportion) of negative pairs in the original data** | **Number (proportion) of negative pairs in the randomized data** |
| --- | --- | --- |
| 1e-7 | 0 | 0 |
| 1e-6 | 0 | 0 |
| 1e-5 | 1 (7.56e-6) | 0 |
| 1e-4 | 3 (2.27e-5) | 0 |
| 1e-3 | 14 (1.06e-4) | 3 (6.62e-8) |
