# Supplementary material for: Co-occurring protein phosphorylation are functionally associated
Source: PLoS Comput Biol. 2017 May 1;13(5):e1005502. doi: 10.1371/journal.pcbi.1005502 (PMC5432191; doi:10.1371/journal.pcbi.1005502)
Supplement: S8 Table — A total of 18,771 high-frequency phosphosites were mapped to known kinase-substrate pairs resulting in 503,077 phosphosite pairs between 284 kinases and 1,797 substrates. (A) Comparison between the positive and negative sets. The positive set is defined as phosphosite pairs in which both sites are located within phosphorylation enriched protein complexes [15]. The negative set is defined as phosphosite pairs in which at least one site cannot be mapped to those complexes. (B) Comparison between the original and randomly permuted data in which the total number of phosphorylations of each protein of the interacting pair at each condition keep fixed. (DOCX) [file pcbi.1005502.s011.docx]

**Number of co-occurring pairs between kinases and substrates across different p-value thresholds.**

**(A)**

| p-value cutoff | Number (%) of co-occurring pairs in the positive set | Number (%) of co-occurring pairs in the negative set | Fold increase  Positive/negative set |
| --- | --- | --- | --- |
| 1E-7 | 169 (0.66%) | 1,983 (0.49%) | 1.35 |
| 1E-6 | 357 (1.40%) | 4,891 (1.21%) | 1.16 |
| 1E-5 | 783 (3.07%) | 11,335 (2.80%) | 1.09 |
| 1E-4 | 1,524 (5.97%) | 25,282 (6.25%) | 0.96 |
| 1E-3 | 2,832 (11.10%) | 50,578 (12.51%) | 0.89 |

**(B)**

| p-value cutoff | Number (%) of co-occurring pairs in the original data | Number (%) of co-occurring pairs in the randomized data | Fold increase  Original/random data |
| --- | --- | --- | --- |
| 1E-7 | 2,414 (0.48%) | 1,287 (0.0010%) | 498.44 |
| 1E-6 | 5,914 (1.17%) | 12,514 (0.0094%) | 124.47 |
| 1E-5 | 13,678 (2.72%) | 90,855 (0.068%) | 40.00 |
| 1E-4 | 30,491 (6.06%) | 565,691(0.42%) | 14.43 |
| 1E-3 | 61,098 (12.14%) | 2,363,794 (1.77%) | 6.86 |
