# Supplementary figures and images for: Co-occurring protein phosphorylation are functionally associated
Source: PLoS Comput Biol. 2017 May 1;13(5):e1005502. doi: 10.1371/journal.pcbi.1005502 (PMC5432191; doi:10.1371/journal.pcbi.1005502)

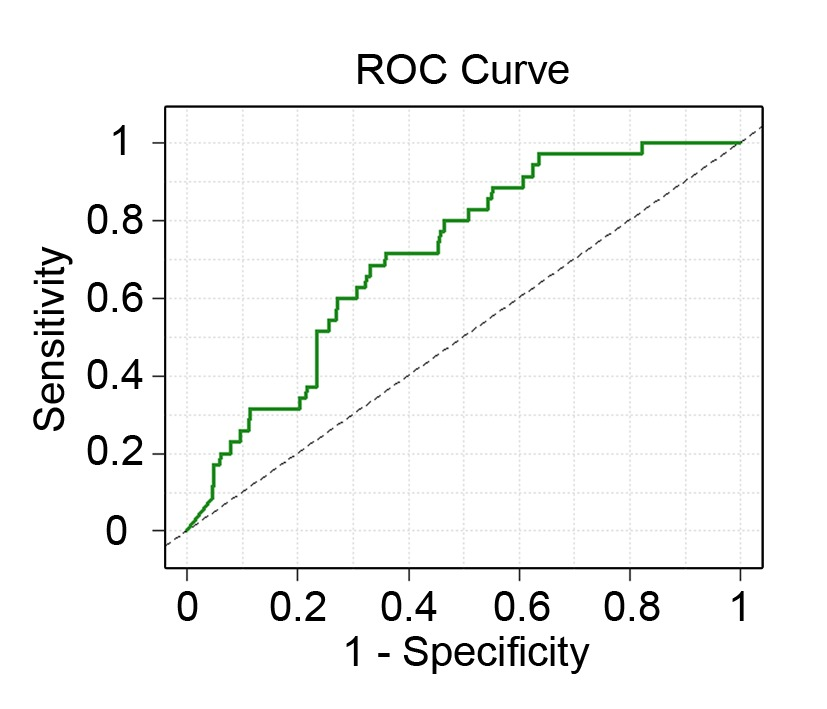

Supplement: S1 Fig — It shows true positives (sensitivity) and false positives (1-specificity) with respect to different FET p-value thresholds. (TIF) [file pcbi.1005502.s012.tif]

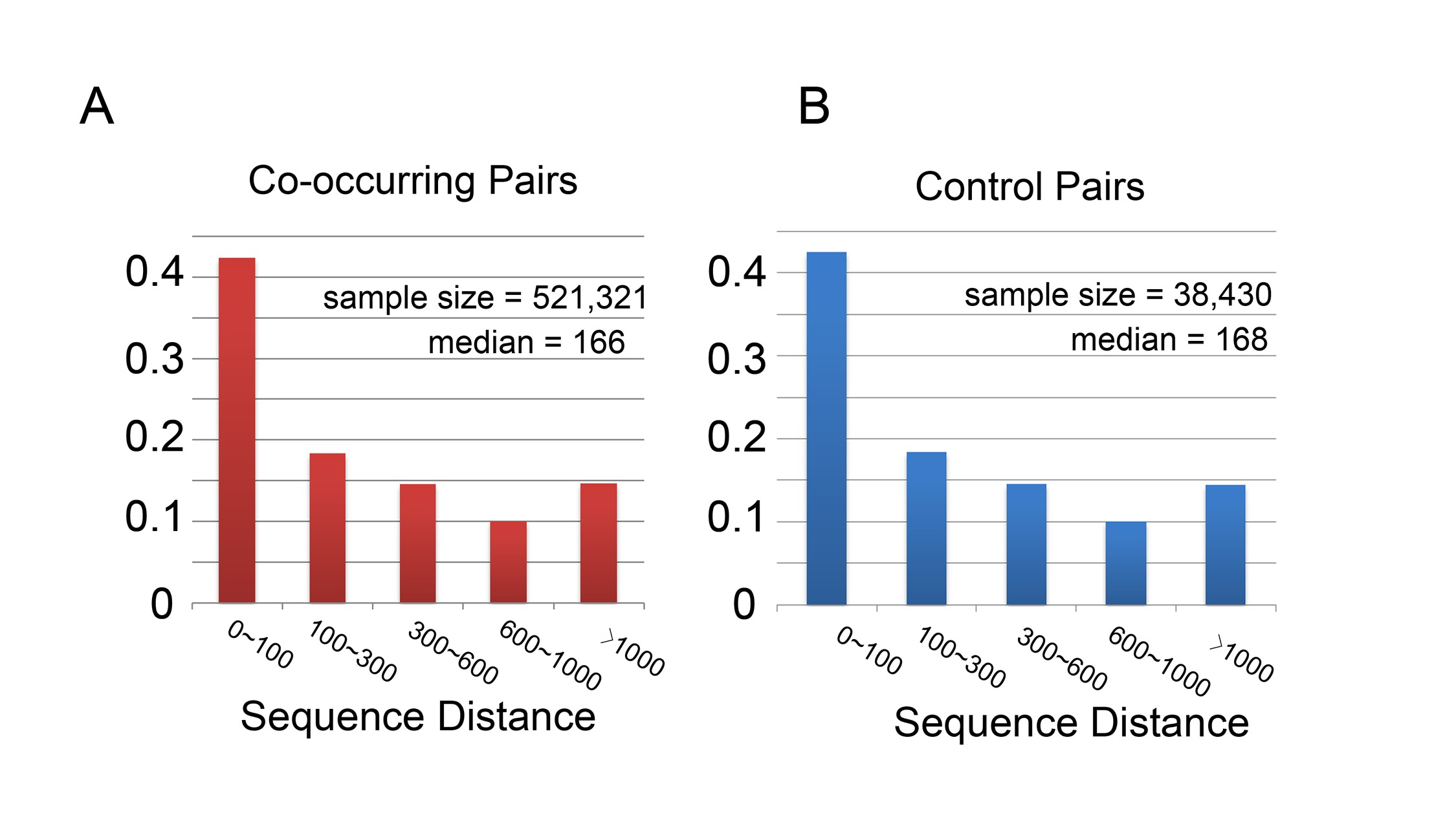

Supplement: S2 Fig — The distribution of sequence distances for the co-occurring pairs (A) and matched control pairs (B) within proteins. (TIF) [file pcbi.1005502.s013.tif]

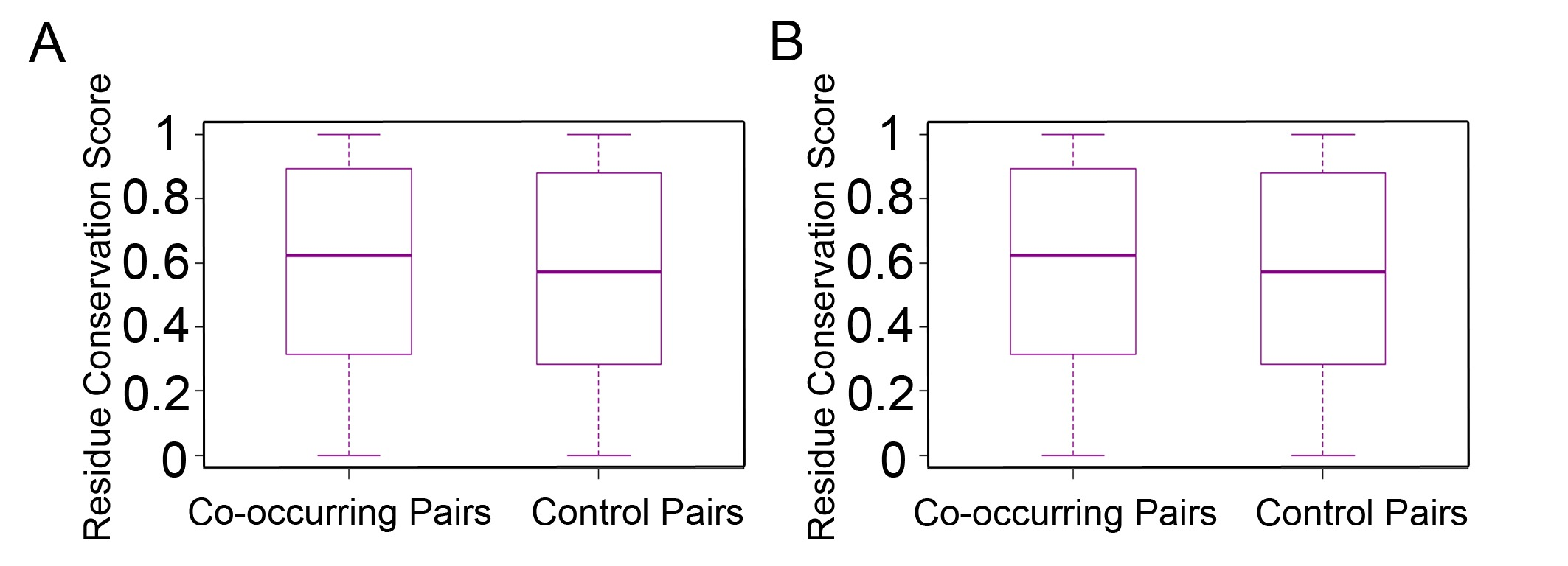

Supplement: S3 Fig — Conservation level is measured by residual conservation score. Comparisons are made using either all phosphosites (A), or after removing sites that are shared by the co-occurring and the control pairs (B). (TIF) [file pcbi.1005502.s014.tif]

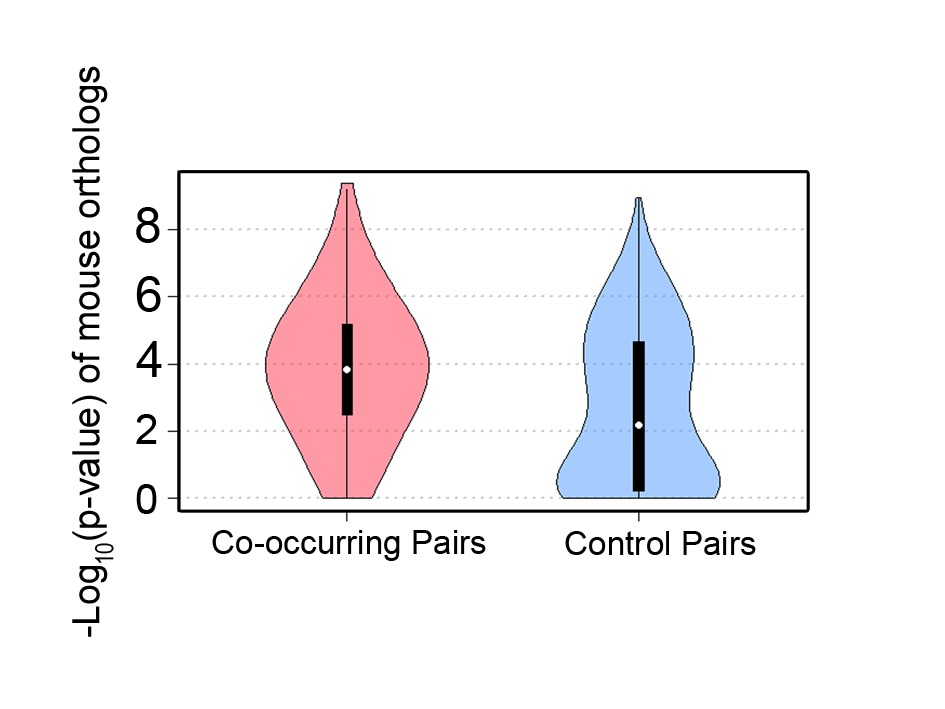

Supplement: S4 Fig — The mouse orthologous phosphosites of human co-occurring pairs are more likely to be modified under the same conditions than those of control pairs. (TIF) [file pcbi.1005502.s015.tif]

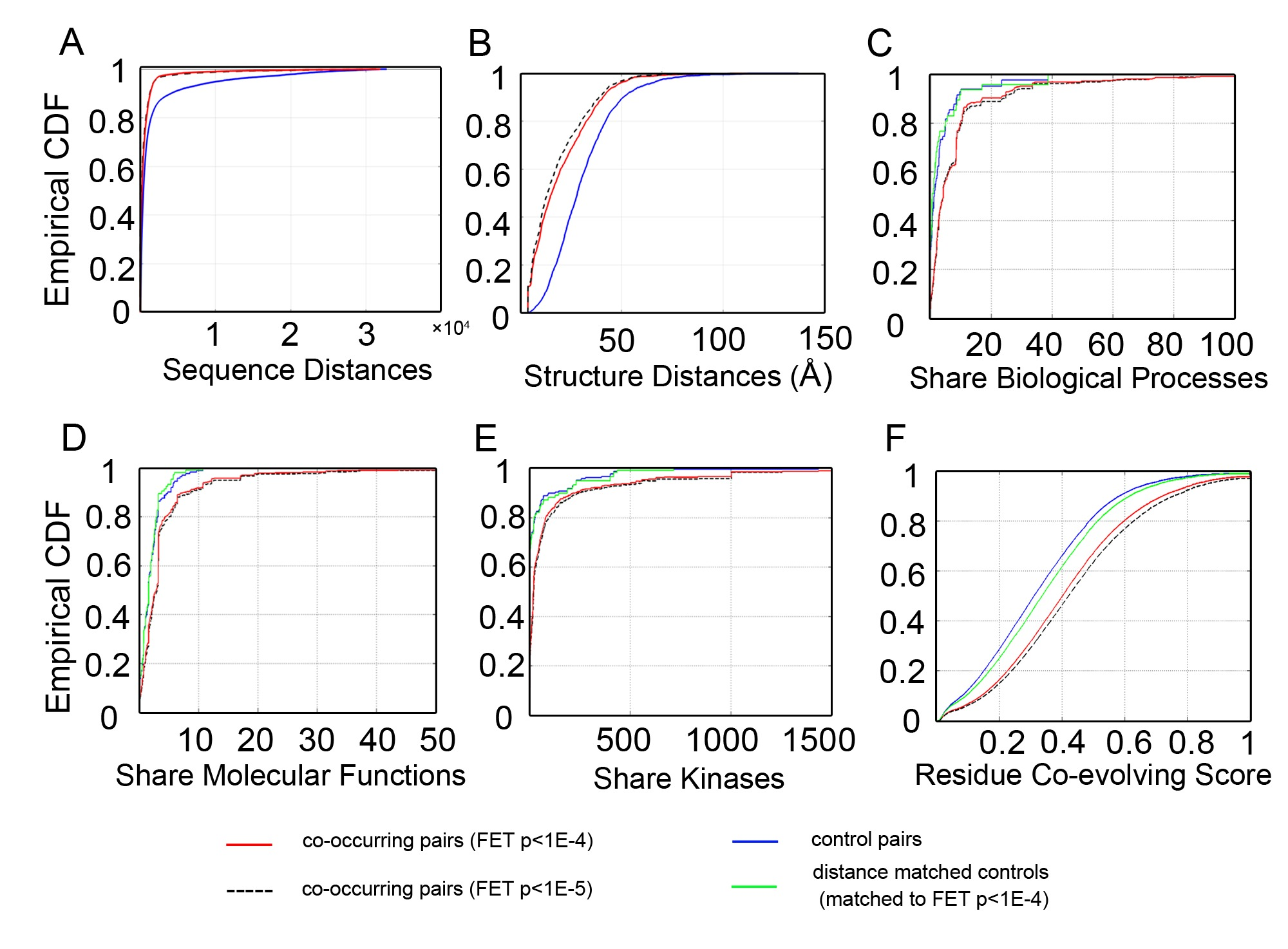

Supplement: S5 Fig — The co-occurring pairs within proteins are defined by FET p-value<1E-4, and controls p-value> = 0.5. The co-occurring and control pairs are compared on their sequence distances (A), 3D structural distances (B), scores that measure sharing annotations of biological processes (C), molecular functions (D), and catalytic kinases (E), and residue co-evolution (nMI) (F). To control for the sequence distance in comparing annotation sharing and co-evolution, co-occurring pairs were also compared with a subset of control pairs with matched distribution of sequence distances. The co-occurring pairs defined by FET p-value<1E-5 are also superimposed on the plots. (TIF) [file pcbi.1005502.s016.tif]

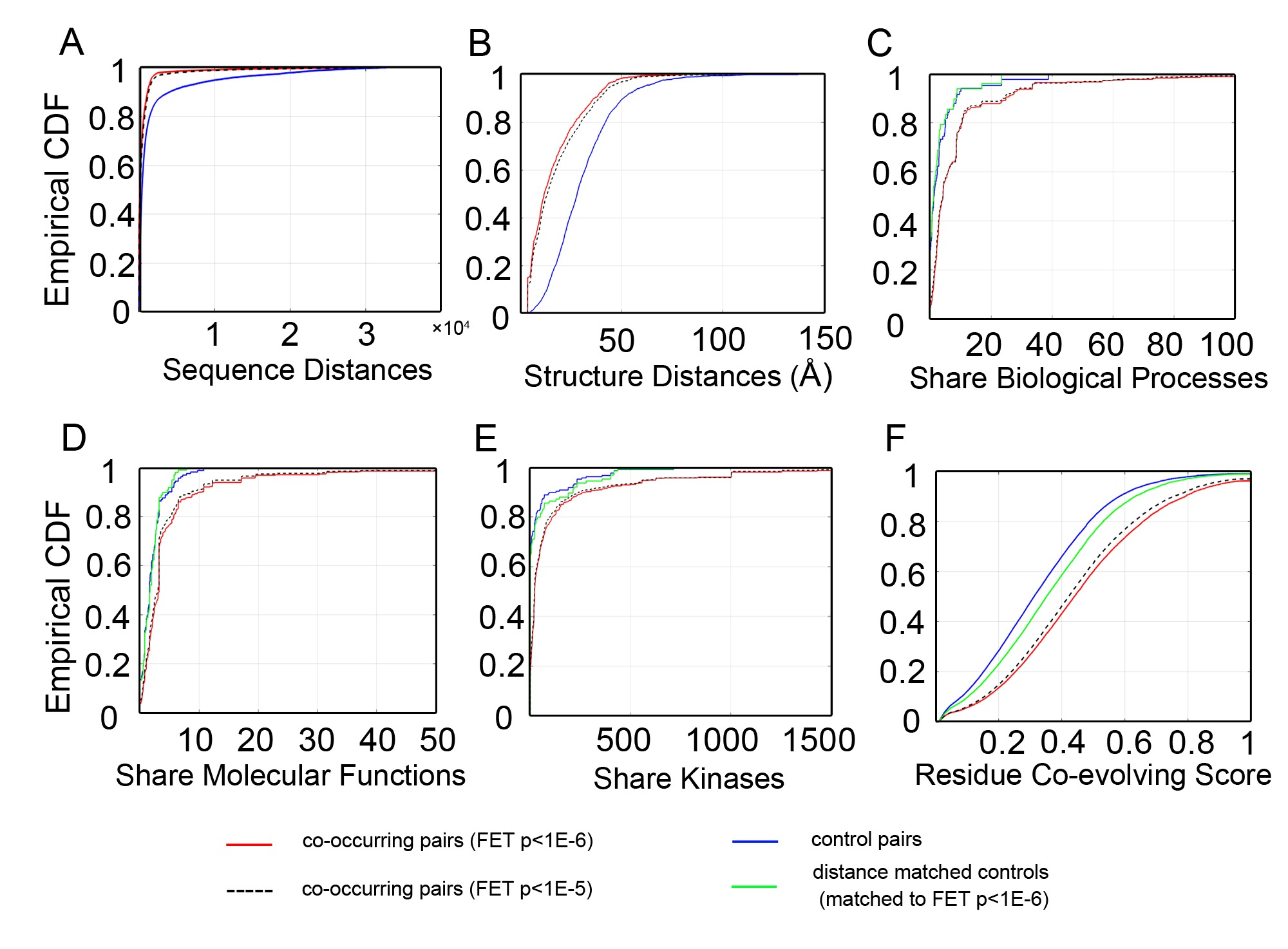

Supplement: S6 Fig — The co-occurring pairs within proteins are defined by FET p-value<1E-6, and controls p-value> = 0.5. The co-occurring and control pairs are compared on their sequence distances (A), 3D structural distances (B), scores that measure sharing annotations of biological processes (C), molecular functions (D), and catalytic kinases (E), and residue co-evolution (nMI) (F). To control for the sequence distance in comparing annotation sharing and co-evolution, co-occurring pairs were also compared with a subset of control pairs with matched distribution of sequence distances. The co-occurring pairs defined by FET p-value<1E-5 are also superimposed on the plots. (TIF) [file pcbi.1005502.s017.tif]

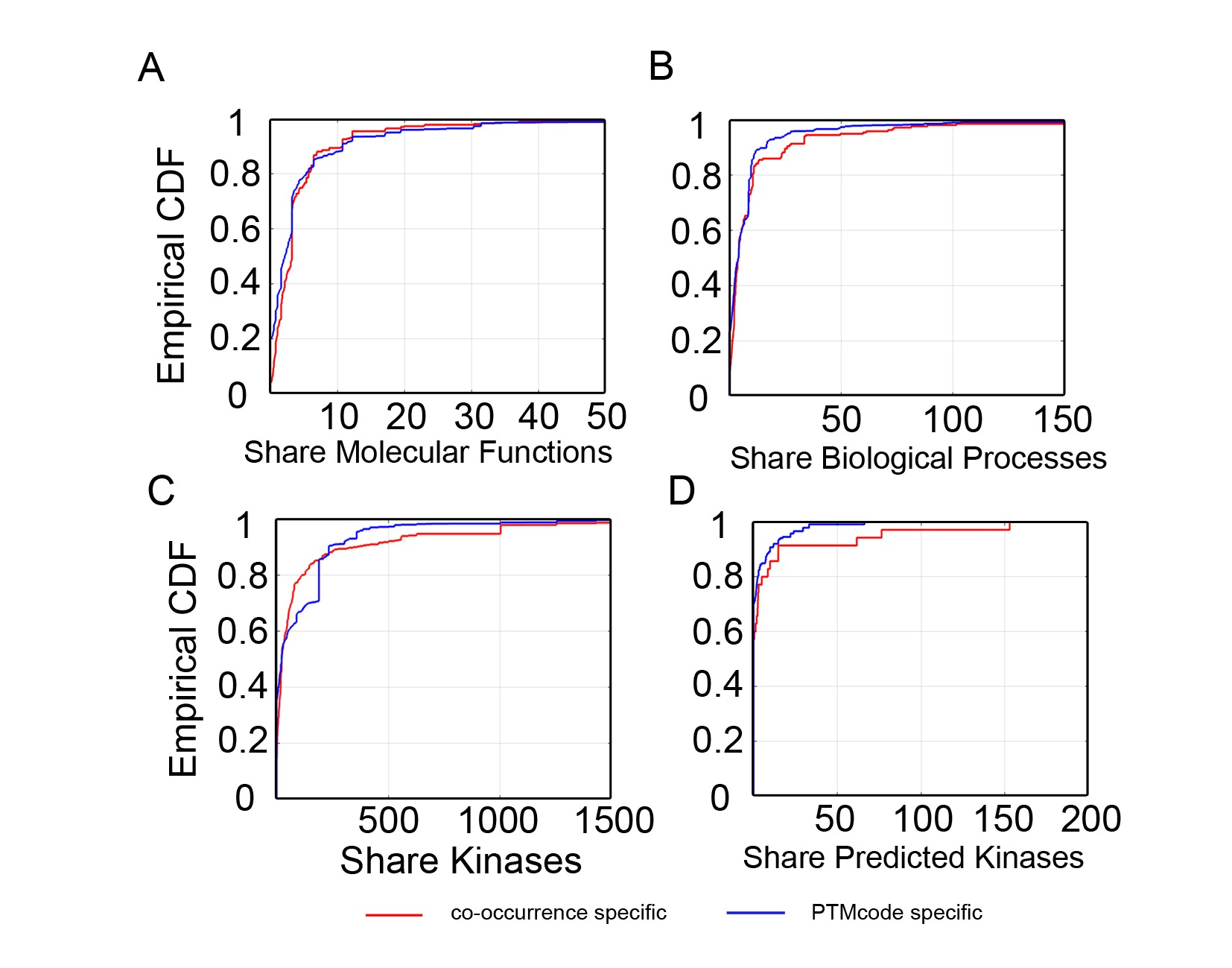

Supplement: S7 Fig — For phosphosite pairs within proteins, pairs that identified only by PTMcode and only from co-occurrence analysis are compared by the scores that measure sharing annotations of molecular functions (A), biological processes (B), and catalytic kinases (C). For phosphosite pairs between interacting proteins, PTMcode specific and co-occurrence specific pairs are compared by the score that measures sharing of computationally predicted kinases (D). (TIF) [file pcbi.1005502.s018.tif]

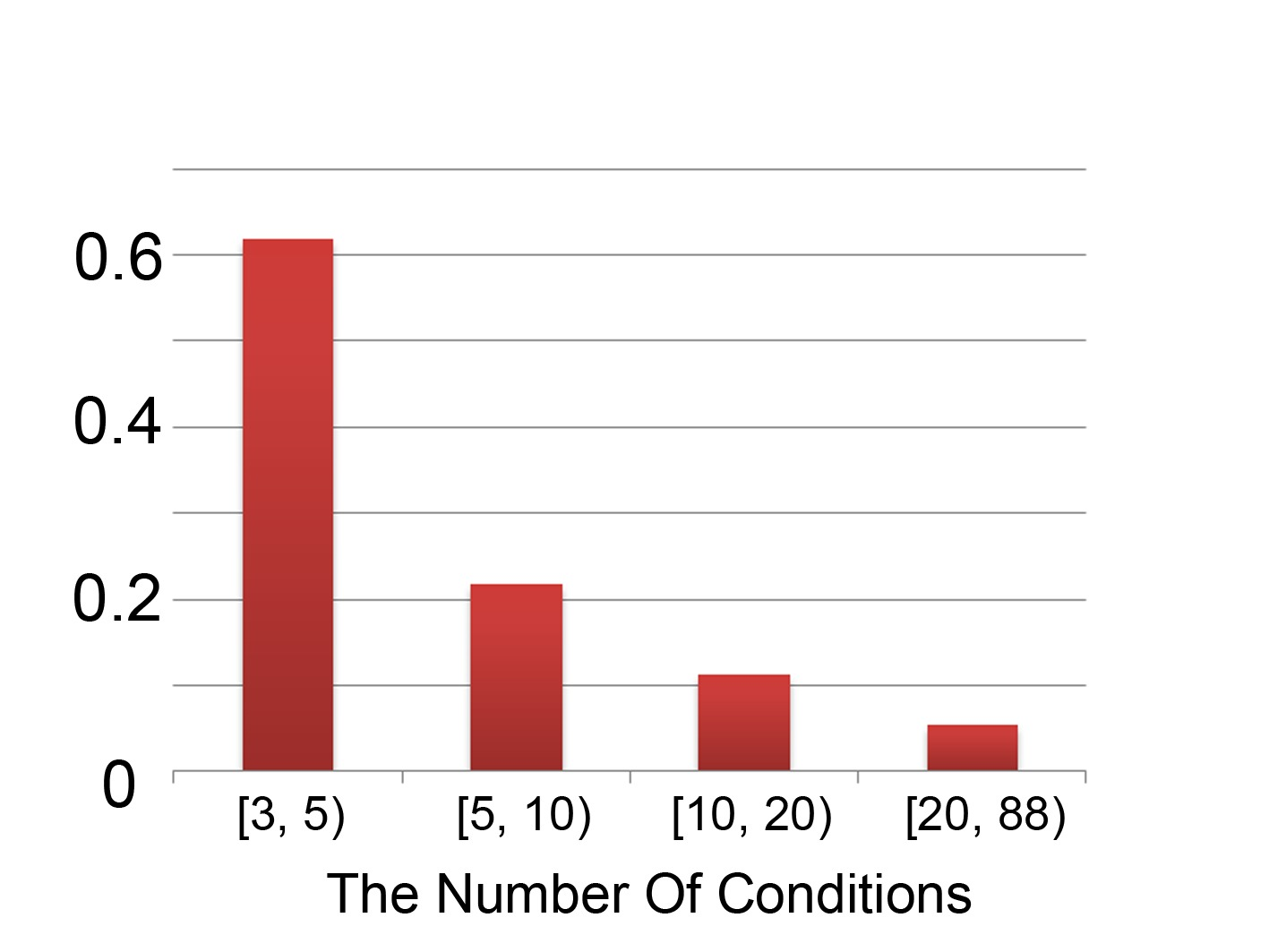

Supplement: S8 Fig — (TIF) [file pcbi.1005502.s019.tif]

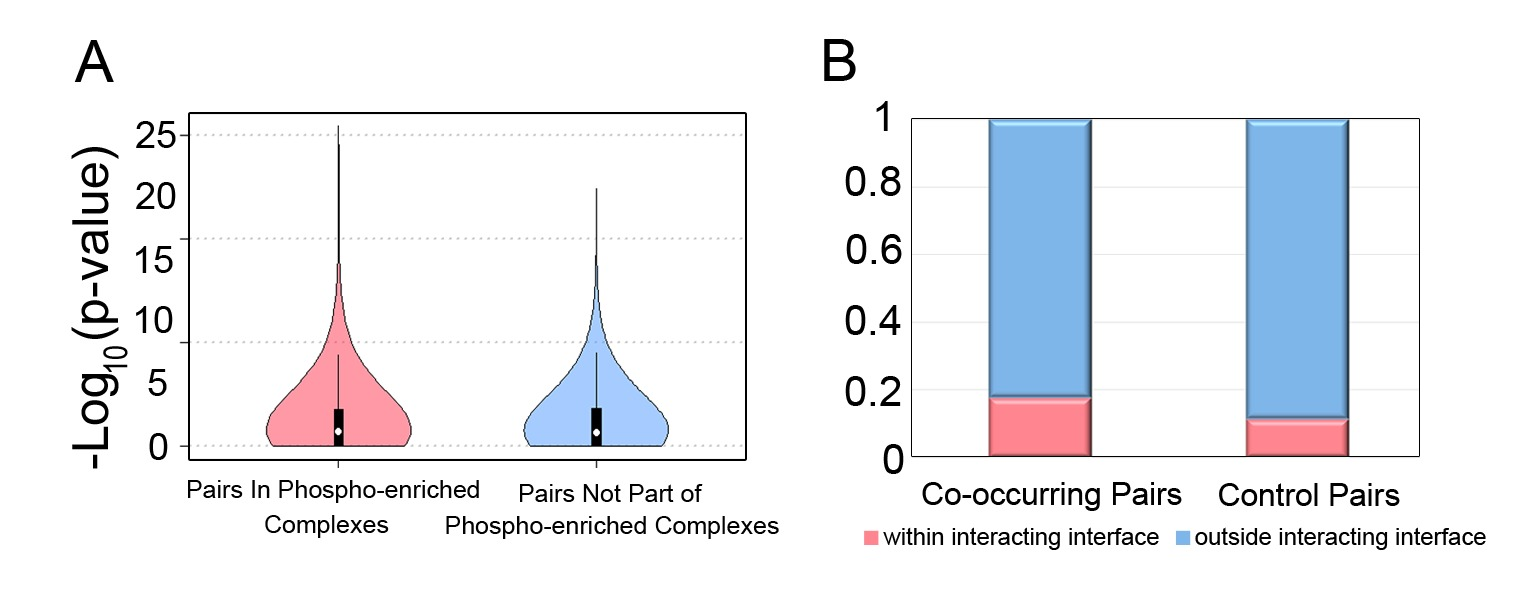

Supplement: S9 Fig — The enrichment of kinase-substrate co-occurring pairs in phosphorylation enriched complexes (A) and in interaction interfaces (B). (TIF) [file pcbi.1005502.s020.tif]
